# Case of Compression of the Brain

**Published:** 1814-07

**Authors:** John Bernard

**Affiliations:** Surgeon of H. M. Sloop the Pelorus


*For the Medical and Physical Journal.*

*Case of Compression of the Brain; by* Mr. JOHN BERNARD.

ROBERT M'CULLUM, æt. 20, November 17th, 1813, in the act of reefing the topsails in a gale of wind at sea, fell from the foretop, and jammed his head with great violence between the gunnels of the forecastle, which intercepted his descent into the water. He was brought down senseless, with two large contused wounds. His head was shaved, and one was apparent over the right orbital process of the frontal bone, extending in a direction upwards, and obliquely, near the cavity formed by the zygomatic process; another on the occipital bone, near the lambdoidal suture. The first wound being considerably enlarged, the pericranium was found detached, and a fracture of about an inch, with a slight depression, was seen; in the other wound I discovered no fracture, but found the pericranium slightly detached.

The man talked incoherently; his pulse was hard and elastic; his skin soon became hot, and he vomited frequently. The hour was about eleven at night, and the brig rolling under the influence of a heavy gale of wind. I contented myself with taking 20 oz. of blood from his arm; when I enlarged the wound, I divided the temporal artery, which then yielded about four ounces. He spent a bad night, slept very little, talked wildly, had frequent vomiting, and two very severe shivering fits; towards morning these symptoms remitted. He was now blooded again, and I should have removed the fractured part, but that the brig rolled and pitched so excessively, as to render a steady position a precarious expectation, exclusive of the symptoms of compression not being very urgent. His pulse was very hard, skin dry, and constricted; he vomited frequently, complained very much of his head, had no sound sleep, and raved with the greatest wildness whenever he dozed; when he was roused, he answered questions rationally, but soon relapsed into a comatose state. He got a purgative enema, which produced two or three motions, and in the evening was let blood again. During night, symptoms of compression became more manifest, and towards morning he had two severe rigors; his vomiting increased, as well as lethargic stupor. The

The wound having the appearance of the letter V, I made an incision in that direction, and with a large trephine removed the fractured part. In making the incision, I divided part of the temporal muscle. He was blooded again, dressed superficially with lint, and a purgative enema administered. During the night he was restless, and complained of his head; but towards morning his pulse became more regular, skin cool, and a gentle diaphoresis broke out; he got at night five grains of calomel, with three of the antimonial powder. On the night of the 21st, he complained of a renewed pain in his head; the wound was dry, the edges of the cutis were loose, flabby, and seemingly detached from the muscles, and altogether the sore bore a very unhealthy aspect; his pulse was quick, and skin very dry. I consulted another surgeon who was in a ship in company, at whose recommendation I took 16 oz. of blood more from the man's arm, which gave him a little ease; but on coming to see him in about half an hour after, I found that having got up to be purged, he fell into a state of deliquium; the lower jaw had fallen, and not feeling any pulsation in his arm, I thought him dying; but on being replaced in bed, and pouring in a little wine, his pulse returned, and he revived. During the night he became restless, but as the morning succeeded all those symptoms remitted. From this period until the 24th, he was under the influence of a hectic fever, and his wound, after excessive suppuration about the scalp and temporal muscle, was assuming an healthy aspect, when a luxuriant fungus of brain shooting through the space vacated by the trephine, induced a severe giddiness and vomiting. I touched it with the argent. nitrat. but on the following morning it rose so considerably, that I was obliged to remove it with a scalpel, and fill up the part with a gradual compression of lint. From this period granulations took place; he met with no unfavourable symptom of much consequence, and though his convalescence was tedious, owing to debility, yet he has now resumed his usual state of health. The wound over the occipital bone healed without any trouble.

*Remarks.*—This case seems to me to be important for three reasons: 1st, The trephine was not applied for 36 hours after the accident; 2dly, A great debility and hectic fever ensued, the symptoms of which were not in the beginning manifest, and were confounded with those of inflammation; and, 3dly, A luxurious fungus of brain, of considerable size, was cut off.

JOHN BERNARD,
*Surgeon of H. M. Sloop the Pelorus.*

